# Structure and Function of the Polycomb Repressive Complexes PRC1 and PRC2

**DOI:** 10.3390/ijms23115971

**Published:** 2022-05-26

**Authors:** Pierre-Olivier Angrand

**Affiliations:** Univ. Lille, CNRS, Inserm, CHU Lille, UMR 9020-U 1277—CANTHER—Cancer Heterogeneity Plasticity and Resistance to Therapies, F-59000 Lille, France; pierre-olivier.angrand@univ-lille.fr

Epigenetic regulation contributes to the control of gene expression programs through local chromatin rearrangements. Amongst the chromatin-regulatory factors, Polycomb group (PcG) proteins are central to epigenetic regulation and chromatin organization. The first PcG gene, *Polycomb* (*PC*), was initially discovered in *Drosophila melanogaster* in 1947 when Pamela Lewis showed that *Pc* mutant larvae displayed an altered segmentation phenotype [1]. Thereafter, other genetic screens identified novel genes whose mutations lead to phenotypes related to the *Pc* loss of function phenotype, thus defining a group of Polycomb (PcG) genes. Yeast two-hybrid experiments as well as biochemical studies have revealed that the PcG proteins assemble to form two main protein complexes called Polycomb repressive complexes 1 (PRC1) and 2 (PRC2) [2,3]. PRC1 and PRC2 are both histone-modifying machines. According to the historical hierarchical model, PRC2 catalyzes the trimethylation of histone H3 at lysine 27 (H3K27me3), whereas PRC1 is recruited at the H3K27me3 mark and monoubiquitinylates histone H2A at lysine 119 (H2AK119ub). These two histone post-translational modifications are then involved in local chromatin compaction and Polycomb-mediated gene repression [4,5].

Originally found in *Drosophila* to be involved in the maintenance of homeotic gene expression control during the fly life cycle [6], PcG genes are evolutionarily conserved and play a role in a plethora of biological processes, including stem cell renewal, the control of cell identity during differentiation and development, and carcinogenesis [7,8,9,10]. In mammalian genomes, most of the PRC1 and PRC2 gene components identified in *Drosophila* exist as multiple orthologs, giving rise to distinct complexes through combinatorial permutations and the mutual exclusion of the subunits within the PRC1 and PRC2. Furthermore, another degree of complexity is added by the differential association of the complexes to various accessory proteins, leading to a tremendous number of complexes having different methods of chromatin recruitment and potentially different biological functions [11].

Due to their involvement in so many fundamental cellular and developmental processes, PRC1 and PRC2 have been the subject of numerous studies over the last 10 years. Recent advances highlight both the complexity of PcG protein assemblies and their biological functions. This Special Issue intends to provide an update on various facets of the relationship between the diversity of the Polycomb repessive complexes PRC1 and PRC2 and their biological outcomes using reviews from different authors.

The review from Geng and Gao focuses on PRC1 protein complexes [12]. It describes the structural complexity of canonical cPRC1 and non-canonical ncPRC1 complexes. Emerging evidence showing that PRC1 also activates transcription under certain circumstances as well as recent studies involving PRC1 in higher-order chromatin organization and chromatin loop formation are discussed in regard to PRC1 subunit composition.

Wang and Wang focus on EZH2, the catalytic subunit of the PRC2 complex [13]. In particular, they detail its non-canonical action on gene and genome regulation. Indeed, beyond its role in Polycomb-mediated repression, EZH2 also exerts biological functions through transcriptional activation instead of repression, the methylation of non-histone targets, scaffolding, and PRC2-independent interaction with a set of transcriptional factors and coactivators.

Varlet et al. [14] emphasize the role played by PRC1 and PRC2 within the plasma cell differentiation process and review the context-dependent action of EZH2, which depends on the humoral immune response phase. In addition, the authors outline the involvement of components of the PRC1 and PRC2 complexes in the multiple myeloma pathophysiology, indicating that targeting PcG proteins in multiple myeloma constitutes a promising therapeutic strategy. In their review, Kaito and Iwana further summarize the involvement of dysregulated PRC functions in hematological malignancies [15]. The authors present the current knowledge on PRC aberrations in myeloid and lymphoid neoplasms together with their prognostic significance and the potential therapeutic approaches. 

Reddington et al. [16] review the role of the Polycomb Repressive-Deubiquinase (PR-DUB) complex, which antagonizes PRC1 action. H2AK119ub is central to Polycomb-mediated gene silencing and while PRC1 attaches this histone modification, PR-DUB removes it. This review highlights the current understanding on the formation and recruitment of the PR-DUB protein complex to chromatin while comparing Drosophila and mammal PR-DUB complexes. Furthermore, the role of PR-DUB in malignant mesothelioma, melanoma, and renal cell carcinoma is outlined, shedding light on a new facet of the involvement of Polycomb repression in oncology.

In conclusion, this Special Issue on “Structure and Function of the Polycomb Repressive Complexes PRC1 and PRC2” provides an overview of the organization of these protein complexes and their roles in physiological and pathological situations, making them potential therapeutic targets in oncology.
